# Applying the vaginal approach for benign ovarian cystectomy: current evidence and future applications

**DOI:** 10.2144/fsoa-2019-0138

**Published:** 2020-04-15

**Authors:** Nicolas Galazis, Stephanie Mappouridou, Srdjan Saso, Konstantinos Lathouras, Joseph Yazbek

**Affiliations:** 1 West London Gynaecological Cancer Centre, Queen Charlotte’s Hospital, Hammersmith Hospital Campus, Imperial College London, Du Cane Road, London W12 0HS, UK; 2 Division of Surgery & Cancer, Institute of Reproductive & Developmental Biology, Imperial College London, Hammersmith Hospital Campus, Du Cane Road, London W12 0HS, UK

**Keywords:** intraoperative ultrasound, vaginal ovarian cystectomy, vaginal pelvic surgery

## Abstract

Vaginal ovarian cystectomy has not gained wide acceptance owing to the potential difficulty in entering the cul-de-sac. We review the current evidence on vaginal approaches to benign ovarian cysts. Outcome measures of interest included time to return to work, patient satisfaction, surgical complications and length of hospital stay. Ten studies were included in this review and involving 525 patients. Vaginal ovarian cystectomy is overall safe and feasible in appropriately selected cases with no evidence of intrapelvic adhesions or endometriosis. These findings will need to be validated in appropriately powered studies, before reliable conclusions can be drawn. Furthermore, we emphasize the importance of ultrasound both preoperatively for case selection optimization and intraoperatively, as a means of guidance during posterior culdotomy.

## Lay abstract

The management of benign ovarian cysts through the vagina (VOC) has not gained wide acceptance by gynecologists worldwide owing to the potential difficulty in accessing the pelvic organs through an incision at the top of the vagina that may cause injury to adjacent structures such as the rectum. This is a review of the literature on the effectiveness of VOC. Various techniques have been described. Overall, VOC is safe and effective in appropriately selected cases. Larger studies will need to be conducted to validate these results. We also emphasize the importance of ultrasound both preoperatively as a tool to select the right patients for VOC as well as during surgery as an adjunct to guide the surgeon.

Vaginal ovarian cystectomy (VOC) refers to the management of ovarian cysts through the vaginal canal [1]. The peritoneal cavity is entered at the pouch of Douglas (POD) or cul-de-sac through an incision at the posterior vaginal fornix allowing access to the pelvic organs [1]. This approach was first described over half a century ago [1] but has not gained wide acceptance by gynecological surgeons. This is secondary to the potential difficulty in entering the peritoneal cavity through the posterior fornix (requiring posterior colpotomy or culdotomy) and its close proximity to the rectum [2]. VOC is not considered the gold standard approach for the management of ovarian cysts and no database exists for clinicians to log their cases for further audit or research analysis. Furthermore, visibility and maneuverability is less when compared with laparotomy or laparoscopy, the latter now being considered the gold standard approach for the management of benign ovarian cysts [2].

Laparoscopic ovarian cystectomy (LOC) is minimally invasive and associated with reduced postoperative pain with a relatively fast recovery period [2,3]. Blind entry laparoscopy using a Veress needle, the preferred entry technique by gynecologists in the UK [4], carries the inherent risk of visceral or vascular injury. General surgeons, by contrast, prefer the open entry technique [5]. Pooled data from 47 randomized trials suggested insufficient evidence to recommend the one entry technique over the other in preventing major visceral or vascular complications [6]. Furthermore, the small abdominal incisions for the insertion of trocars are associated with wound infection, herniation and visible scarring [2,3].

Due to the potential risks associated with LOC, which is driven by a need for alternative minimal access procedures that are economic and is associated with short hospital stay, the interest in VOC has recently been revived. Obviating the need for skin incisions or CO_2_ insufflation renders the vaginal approach an attractive alternative option. Over the past decade, a number of studies have been conducted investigating the feasibility of VOC and comparing it with LOC. Few techniques of VOC have been described, with attention to the initial entry in the peritoneal cavity via a posterior culdotomy. This step that can be associated with adverse outcomes secondary to the close proximity to the rectum, particularly in cases of extensive adhesions or endometriosis.

A hybrid vaginal approach for ovarian cystectomy includes natural orifice transluminal endoscopic surgery (NOTES). This combines the benefits of both conventional vaginal and laparoscopic approaches as it avoids visible abdominal scars and allows better visualization and access to the abdominal cavity when compared with conventional VOC [7]. The main challenge of vaginal NOTES is associated with the restriction and conflict between the instruments during single-port surgery [7].

When comparing the different approaches to ovarian cystectomy, the findings are inconsistent when it comes to operating times (OT), rate of cyst spillage, length of hospital stay and degree of postoperative pain. To date, no systematic review has been conducted to clarify the matter. The aim of our study is to critically appraise the evidence and evaluate the feasibility and safety of ovarian cystectomy through the vaginal route, including simple VOC, transvaginal NOTES and other mixed laparoscopic and vaginal approaches. Our secondary aim is to propose a safe, easy and widely accepted step-by-step technique of VOC.

## Methods

### Search strategy for the identification of eligible studies

Institutional review board approval was not necessary as this review did not require any patient identifying information. The systematic search followed PRISMA guidelines [8]. A bibliographic search of English language publications in three computerized databases (PubMed, Science Direct and SciFinder) was conducted. The search was augmented by a snowball strategy, examining the references cited in primary sources and review manuscripts.

### Study selection

We reviewed studies where benign ovarian cysts were managed using a vaginal approach. Only studies published in English and in peer-reviewed journals were considered. Because of the scarcity of studies on this topic, any study design was deemed suitable for consideration including nonrandomized and observational studies. Review articles were excluded. There were no exclusions in relation to the country of origin of the study or the date of publication.

**Figure 1.**
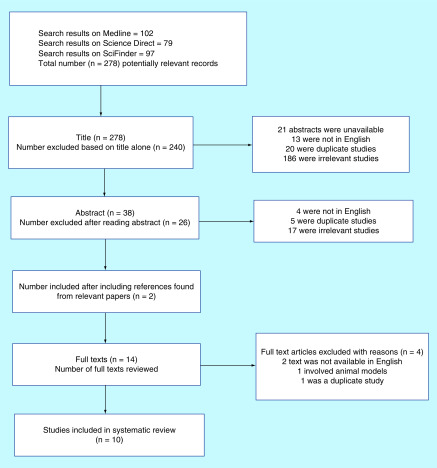
Flow chart of the study selection.

The following search terms have been used in all three electronic databases (Figure 1):

- (transvaginal surgery) AND (ovarian cyst);
- (vaginal ovarian cystectomy);
- (vaginal) AND (benign ovarian cyst),
- (natural orifice transluminal surgery OR NOTES) AND (ovarian cystectomy);
- (natural orifice transluminal surgery OR NOTES) AND (benign ovarian cyst);
- (natural orifice transluminal surgery OR NOTES) AND (gynecology).

The search was conducted by the first author (N Galazis), repeated independently by a second author (S Mappouridou) and cross-checked by a senior author (S Saso). This took place in the first week of September 2019, therefore studies published after this date have not been screened. Our group has conducted numerous literature reviews, therefore we did not seek the assistance of a research librarian for the literature search. The above are summarized in Table 1.

**Table 1.** Search and selection strategy for the systematic review of vaginal approaches to benign ovarian cysts.

Databases searched	PubMed, Science Direct, SciFinder
Search keywords	– (transvaginal surgery) AND (ovarian cyst) – (vaginal ovarian cystectomy) – (vaginal) AND (benign ovarian cyst) – (natural orifice transluminal surgery OR NOTES) AND (ovarian cystectomy) – (natural orifice transluminal surgery OR NOTES) AND (benign ovarian cyst) – (natural orifice transluminal surgery OR NOTES) AND (gynaecology)
Other sources	Additional studies were identified through references of included studies and reviews
Inclusion criteria	– Articles written in English and published in peer-reviewed journals – Any study including randomized, nonrandomized and case–control studies and case reports/series
Exclusion criteria	– Papers not in English – Full articles not available – Studies on animal models – NOTES in nongynecological or nonovarian surgery – Review articles

The initial search, using the search terms described above, identified a total of 278 records (Medline n = 102, Science Direct n = 79, SciFinder n = 97), where 48 were duplicates. These have been published between 1966 and 2017.

### Data extraction

A data extraction spreadsheet was developed and agreed between the authors. The selected studies were comprehensively examined. Relevant data were extracted for each paper and inputted into the spreadsheet by the first author (N Galazis) and subsequently cross-checked by the second author (S Mappouridou). The information selected included author details, year of publication and country of the study, study aim, sample size, methodology, sample characteristics, outcome measures and conclusions. Disagreements regarding extracted data were resolved by discussion and deliberated on by the most senior author (S Saso). Tables 2 and 3 list the main characteristics of the selected studies.

**Table 2.** Patient characteristics.

Study (year) and institution	Participant groups (N)	Selection criteria	Demographics	Ref.
1. Yoong *et al*. (2016) North Middlesex Hospital, London, UK	Patients who underwent primary ovarian cystectomy for a nonmalignant cyst, n = 49; Vaginal approach (n = 28), Laparoscopic approach (n = 21)	Inclusion: Nonmalignant ovarian cyst on TVUS, CA 125<35 iu, absence of intrapelvic adhesions in POD confirmed during examination under anesthesia on the day	Not specified, however, no statistical difference in mean age, parity and BMI between the two groups	[5]
2. Tanaka *et al*. (2008) Kanazawa University and Sagawa Clinic, Kanazawa, Japan	Patients with benign ovarian cysts located in the cul-de-sac, who underwent VOC (n = 16; 14 with unilateral cysts and 2 with bilateral cysts)	Exclusion: Cyst outside the cul-de-sac, severely adhesive or suspected of being malignant	Mean age: 33 (22–50) Four women were Nulliparous	[9]
3. Yoshiki *et al*. (2012) Tokyo Medical and Dental University, Tokyo, Japan	Patients that underwent hybrid transvaginal and laparoscopic adnexal surgery (n = 15) (salpingo-opherectomy n = 7, cystectomy for ovarian tumors, n = 3 and salpingectomy for unruptured tubal pregnancy, n = 5)	Not specified	Mean age: 41 (32–50) BMI: 22.1 (17.5–27.5)	[10]
4. Tanaka *et al*. (2012) Kanazawa University and Sagawa Clinic, Kanazawa, Japan	Laparoscopic cystectomy (n = 40) Vaginal approach (n = 35)	Inclusion: Radiologically unilateral benign cysts Exclusion: Bilateral cysts, cysts outside cul-de-sac, elevated tumor markers	Vaginal group: Age: 31.5 (±6), BMI: 20.8 (±3), Cyst diameter (cm): 6.1 (±2.0), Nullipara: 20/35 (56%) Laparoscopy group: No statistical difference in the above demographics compared with the vaginal group	[11]
5. Tanaka *et al*. (2013) Kanazawa University and Sagawa Clinic, Kanazawa, Japan	Questionnaire to patients who had undergone ovarian cystectomy using a transvaginal approach (n = 73)	Inclusion: Radiologically unilateral benign cysts	Mean age: 33 (±6.1), 64% were multigravidas, 45% were pluripara	[12]
6. Wang *et al*. (2016) Chang Gung Memorial Hospital, Linkou, Taiwan	Patients with presumed benign ovarian cysts (n = 277) NAOC group (n = 34) compared with LOC (n = 243)	Inclusion: Ovarian masses clinically diagnosed of low probability of malignancy on TVUS or CT and CA125 <65 iu Exclusion: Suspected adhesions, suspected severe endometriosis with obliteration of POD during clinical examination	NAOC group: 33.6 ± 6.4 (21–48), BMI: 21.8 ± 3.0 (17.0–28.9), Cyst diameter (cm): 7.6 ± 1.8 (5–12), Nulliparae: 18 (53%) LOC group: No statistical difference between the two groups in the above parameters	[13]
7. Wang *et al*. (2009) Chang Gung Memorial Hospital and University College of Medicine, Tao-Yuan, Taiwan	Patients of reproductive age with prior sexual activity and clinically diagnosed benign large ovarian masses (10–27 cm), n = 10	Inclusion: Large, benign ovarian masses. Nonmalignant characteristics on TVUS and CT, CA125 <65 iu	Median age: 29 (18–35), BMI: 22.1 (18.5–27), Nulliparous: 6, Multiparous: 4	[14]
8. Ding *et al*. (2017) Buddhist Tzu Chi General Hospital, Hualien, Taiwan	Patients undergoing NOTES VH (n = 4) or OC (n = 2) between September 2009 to December 2009 (n = 6)	Inclusion: Nonvirgins, no previous pelvic infection or obliteration of POD	Patient 1 – Age: 46, BMI: 20.3, Cyst (cm): 4.6, Parity: 1 Patient 2 – Age: 24, BMI: 29.9, Cyst (cm): 5, Parity: 0	[15]
9. Tanaka *et al*. (2011) Kanazawa University, Kanazawa, Japan	Nonvirgin patients undergoing ovarian cystectomy (n = 36)	Inclusion: Nonvirgins, benign ovarian cyst on TVUS or MRI, located in the cul-de-sac, absence of adhesions on TVUS and pelvic examination *Exclusion*: Teratomas with raised aFP levels	Mean age: 32 (± 5.7), BMI: 20.7 (±3.1), 23/36 Nulliparous, Cyst (cm): 6.9 (±2.1)	[16]
10. Baekelandt (2017) Imelda Hospital, Bonheiden, Belgium	Patients who consented for transvaginal treatment of benign ovarian cyst (n = 14)	Inclusion: Benign ovarian cyst on TVUS	Not specified	[17]

BMI: Body mass index; CA125: Cancer antigen 125; CT: Computed tomography; LOC: Laparoscopic ovarian cystectomy; NAOC: Natural orifice transluminal endoscopic surgery-assisted ovarian cystectomy; NOTES: Natural orifice transluminal endoscopic surgery; OC: Ovarian cystectomy; POD: Pouch of Douglas; SD: Standard deviation; TVUS: *Trans*-vaginal ultrasound; VH: Vaginal hysterectomy; VOC: Vaginal ovarian cystectomy.

**Table 3.** Main characteristics of the studies.

Study (year) and institution	Aim of study and intervention	Study design	Surgical technique	Outcome measures	Results	Conclusions	Ref.
1. Yoong *et al*. (2016) North Middlesex Hospital, London, UK	To compare surgical outcomes, cost–effectiveness and patient satisfaction in patients undergoing vaginal (VOC) or LOC for benign ovarian cysts	Retrospective case–control study	VOC: Dorsal lithotomy, Vulsellum to posterior lip of cervix, local anesthetic with adrenaline to posterior fornix, transverse culdotomy, POD digitally checked, incision enlarged to 3 cm, Sims speculum inserted to reflect rectum posteriorly, cyst capsule grasped and exteriorized, routine cystectomy performed. Large cysts aspirated prior to exteriorization and cystectomy	Duration of surgery, intraoperative complications, EBL, length of inpatient stay, postoperative pain, cost of surgical procedure, patient satisfaction scores, overall cost–effectiveness	VOC compared with LOC: Mean operating time: 91.7’ vs 78’, p < 0.001; EBL: 116 vs 95 ml, p < 0.001; Intraoperative spillage: 6 vs 35%, p < 0.001; length of stay: 10.9 vs 8.9 h, p < 0.001; pain score: 2/10 vs 4/10, p < 0.05; patient satisfaction: 8.2/10 vs 6.5/10, p < 0.001; Cost: £1690 vs 1760, days to return to work: 13.6 vs 39.2, p < 0.001; loss of income and productivity due to delay eδ return to work: £648 vs £1869. Complications: VOC: x1 rectal injury in a case of severe endometriosis with obliteration of POD requiring laparotomy and defunctioning colostomy. X1 conversion to laparoscopy for endometrioma LOC: No immediate complications, x1 case small bowel obstruction at lateral port site requiring readmission and surgery, x2 superficial wound infections managed on oral antibiotics	VOC took longer to perform and leads to longer inpatient stay, however, patients had less postoperative pain and higher satisfaction. VOC is viable and cost effective in appropriately selected cases	[5]
2. Tanaka *et al*. (2008) Kanazawa University and Sagawa Clinic Kanazawa, Japan	To evaluate feasibility of US-guided culdotomy using a renal balloon dilator catheter for transvaginal ovarian cystectomy	Interventional, nonrandomized study	Enema administered the day before. Dorsal lithotomy. TVUS with an adapter for puncture inserted into the vagina. Cyst puncture under US-guidance with a 19-G needle via posterior fornix. Inner needle extracted and replaced with a guide wire under US guidance. TVUS probe withdrawn and Nephromax balloon dilator catheter passed over the guide wire and inflated. Balloon and guide wire withdrawn and culdotomy enlarged with forceps. Ovarian cyst wall located through culdotomy and partially exteriorized. Contents aspirated with a needle and cyst exteriorized and decapsulated. Culdotomy closed with sutures	Success of the procedure, operating time, blood loss, complications	Success of procedure: 15/16 (94%) Mean ± SD operating time for culdotomy was 22 ± 11 min. Blood loss was <10 ml. Mean ± SD CRP value on postoperative day 3: 1.98 ± 1.39 mg/dl. No intraoperative complications including rectal injury No patient developed fever beyond postoperative day 3. Unsuccessful procedure: one woman culdotomy failed because the pressure of the puncturing needle pushed the dermoid cyst out of the cul-de-sac.	Under US-guidance with the assistance of a dilator culdotomy is safe and reliable	[9]
3. Yoshiki *et al*. (2012) Tokyo Medical and Dental University Tokyo, Japan	To evaluate the feasibility, safety and operative outcomes of hybrid transvaginal and laparoscopic adnexal surgery	Retrospective data analysis study	Lithotomy position, open laparoscopic entry through umbilicus and pneumoperioneum inflated to 10 mmHg. Two 5 mm trocars inserted through posterior fornix of under laparoscopic guidance. Procedure performed using a 5-mm scope though umbilicus and graspers through vaginal trocars. Specimen retrieved with endopouch. Culdotomy closed with sutures	Operative time, blood loss, need for conversion to open or multiport laparoscopic surgery, gynecologic examination findings 3 months later and cosmetic result	Mean operative time: 79 min (range: 49–116) Blood loss: <10 ml All procedures were successful, no conversion to conventional multiport laparoscopy or open surgery. No peri- or postoperative complications recorded during recovery or in the 3 months’ follow-up	Hybrid transvaginal and transumbilical laparoscopic surgery is safe and feasible in select patients	[10]
4. Tanaka *et al*. (2012) Kanazawa university and Sagawa Clinic Kanazawa, Japan	To evaluate the feasibility of VOC and using US-guided culdotomy and laparoscopic back-up, and compare it with LOC in patients with dermoid cyst	Retrospective review	Enema administered the day before. Dorsal lithotomy. Culdotomy assisted by TVUS along with either a renal balloon dilator catheter (n = 8) or umbrella Hakko needle (n = 27). Balloon dilator technique: Centesis to the vaginal wall by the needle under US-guidance, balloon catheter dilated the route toward cul-de-sac Umbrella Hakko needle (UHN): TVUS probe with a needle guide inserted into the vagina and directly into the cyst. Umbrella portion of UHN opened once in the cyst. TVUS probe withdrawn. Culdotomy enlarged to 3 cm. Ovarian cyst exteriorized and if necessary aspirated or culdotomy extended. Routine cystectomy performed and repair of culdotomy	Completion rate and conversion to laparoscopy or laparotomy, intraoperative complications, operating time, hemoglobin (Hb) decrease on postoperative day 1, CRP level on day 3 and postoperative complications	Vaginal approach compared with Laparoscopic: Nulliparus: Operating time, median (IQR): 95.5 (74–129) vs 120 (96–140; p = 0.15) Hb decrease in g/dl; mean ± SD: 1.65 ± 0.55 vs 1.61 ± 0.90 (p = 0.87) CRP day 3; median (IQR): 1.5 (0.6–2.6) vs 1.9 (0.4–3.2; p = 0.22) Multiparus: Operating time, median (IQR): 80 (63–126) vs 105 (90–131; p = 0.19) Hb decrease in g/dl; mean ± SD: 1.43 ± 0.82 vs 1.63 ± 0.81 (p = 0.54) CRP day 3; median (IQR): 1.8 (1.1–2.6) vs 1.9 (1.3–2.6; p = 0.26) All cystectomies (n = 75) were completed without conversion to laparotomy One conversion to laparoscopy in the vaginal group due to uncontrolled bleeding No major intraoperative complications	Vaginal ovarian cystectomy using US and an umbrella needle showed a reliable profile comparable to conventional laparoscopy for the treatment of dermoid cyst located in cul-de-sac. The method may be preferred for minimally invasive surgery	[11]
5. Tanaka *et al*. (2013) Kanazawa University and Sagawa Clinic Kanazawa, Japan	To assess long-term complications, including infertility and dyspareunia after transvaginal peritoneal surgery	Questionnaire survey	Anonymous questionnaires sent to patients had VOC more than 6 months earlier, between 2003–2011 60% of questionnaires returned (n = 44)	Obstetric history before or after surgery (fertility), gynecological symptoms before or after surgery (dyspareunia, abnormal vaginal bleeding, dysmenorrhea) Patient satisfaction (5-point scale)	Fertility: 38% (9/24) of the patients that did not use contraception conceived (total of 12 pregnancies) (mean postoperating period 18.3 months, SD: 9.1) Five normal vaginal deliveries, no cesarean section before or after transvaginal cystectomy Gynecological discomfort: At 1 month postoperation: 5% complained of dyspareunia and 5% postcoital bleeding (all episodes resolved at 2 months) Three patients with endometriomas (7%) complained of dysmenorrhea postoperation for at least 1 year Satisfaction: 4.12/5	Transvaginal surgery (with adequate culdotomy) does not interfere with fertility and does not cause dyspareunia. Dysmenorrhea maybe reduced, except in the cases of endometriomas. Many patients find it satisfactory	[12]
6. Wang *et al*. (2016) Chang Gung Memorial Hospital Linkou, Taiwan	To examine the safety and efficacy of NAOC for benign ovarian tumors	Retrospective case-matched study	Dorsolithotomy position, posterior colpotomy incision extended by digital pressure. A surgical glove applied to a vaginal wound retractor. Three sheaths (one 10 mm and two 5 mm) were inserted through the ‘finger tips’ and tied with elastic bandages to prevent desufflation of pneumoperitoneum. Camera and laparoscopic instruments were inserted. Ovarian cyst pulled into POD and glove detached. Wound retractor still in place. Under direct vision cyst was drained, exteriorized to vagina for ovarian cystectomy. Glove reattached and pneumoperitoneum reestablished to inspect and ensure hemostasis. Colpotomy incision was sutured at the end	Clinical outcomes: EBL, perioperative complications Efficiency outcomes: Operating time, postoperative stay and total hospital charges	EBL: 31.62 ± 24.04 (5–100) vs 21.41 ± 14.75 (5–50), p = 0.028 Febrile morbidity: 0 vs 1 (p = 1) Operating time (min): 38.12 ± 10.19 (29–65) vs 53.82 ± 18.61 (30–120) (p < 0.001) Postoperative stay (days): 1.38 ± 0.55 (1–3) vs 1.82 ± 0.52 (1–3; p < 0.001) Hospital charges (£): 255.95 ± 74.85 (118.51–435.02) vs 290.87 ± 116.88 (95.84–688.12; p = 0.698) All procedures completed successfully with no conversion to open or laparoscopic (in NAOC group) No major complications noted No abnormal findings or recurrence noted at 6 months follow-up	NAOC for presumed benign and large tumors (up to 12 cm) is safe and possible in well-selected patients. NAOC offers superior operative efficiency compared with LOC	[13]
7. Wang *et al*. (2009) Chang Gung Memorial Hospital and University College of Medicine, Tao-Yuan, Taiwan	To present experience with LAVOC for large ovarian masses	Retrospective study	Dorsal lithotomy position. Routine laparoscopic entry through umbilicus. Controlled drainage of cyst under direct vision. Pneumoperitoneum created. Two more laparoscopic ports inserted. Transverse colpotomy incision using unipolar scissors. Collapsed ovarian mass pushed into the cul-de-sac. CO_2_ insufflator and videolaparoscopic system switched off. Colpotomy incision extended digitally. Heaney retractor used to depress rectum. Mass exteriorized in the vagina. Decapsulation of cyst off healthy ovarian tissue. Colpotomy incision was closed. Pneumoperitoneum re-established. Irrigation of peritoneal cavity before closure	Successful completion of procedure, operative time, EBL, mass diameter, postoperative stay, intra- and postoperative complications	Mean operative duration is 62 min, EBL, median (range): 50 ml (10–150) Median diameter of masses: 15.5 cm (range: 10–27) Postoperative hospital stay; median: 2 days (range 1–4) – <48% in 90%. No gross intra-abdominal spillage Median aspirated fluid 1000 ml (range: 800–3500 ml) No open conversion No major complications (ureter or bowel injury, postoperative pelvic abscess or blood transfusion required) No abnormal findings or mass recurrence at 6-month follow-up. One case of postoperative low-grade fever controlled by intravenous antibiotics	In careful selection of patients with adequate vaginal capacity and low malignant probability of the mass, LAVOC can be performed without difficulty in dealing with large ovarian tumor. Thereby avoiding laparotomy and intraperitoneal tumor content spillage	[14]
8. Ding *et al*. (2017) Buddhist Tzu Chi General Hospital, Hualien, Taiwan	To report initial experience with patients undergoing *trans*vaginal NOTES using single-port technique in benign gynecological disease	Retrospective data analysis study	Ovarian cystectomy: Dorsal lithotomy position. Posterior colpotomy was performed. Wound retractor was inserted with a surgical glove attached. Three cannulas inserted into cul-de-sac through glove fingers (one 10 mm and two 5 mm trocars). Pneumoperitoneum created. A 30° endoscope and two conventional 5-mm laparoscopic instruments inserted into pelvic cavity. Decapsulation of cyst followed by exteriorization by detaching the glove through colpotomy wound. Surgical glove re-attached to inspect for bleeding. Colpotomy sutured	Surgical outcomes: Blood loss, operating time, fever, hospital stay, visual analog pain scores at 2, 24 and 48 h postoperatively, and clinic follow-up in 1 week, 1 and 6 months	No conversation to laparoscopy or laparotomy. No blood transfusions required. Pain score of 0 at 48 h postoperation. Good healing of vaginal cuff on follow-up examinations OC (n = 2): Mean surgical time: 74 min, EBL: 50 ml, mean hospital stay: 3.5 days, Pain score: 0 at 48 h	NOTES surgery is feasible and safe in patient with benign pathology of the ovary and uterus. It has the advantage of reduced pain and better cosmetic outcomes	[15]
9. Tanaka *et al*. (2011) Kanazawa University Kanazawa, Japan	To evaluate feasibility of an US-guided culdotomy using newly developed umbrella needle (umbrella Hakko needle technique: Culdotomy 2U)	Interventional nonrandomized trial	Dorsal lithotomy position. TVUS with a needle guide inserted. Cyst directly punctured under US guidance with an umbrella Hakko needle under US guidance. Umbrella portion opened and stabilized with forceps. Inner needle inserted and US probe with needle guide extracted while needle remains, penetrating the center of the posterior fornix. Needle retracted gently, vaginal walls incised on both sides with an electric scalpel. Cyst becomes visible and partially exteriorized with gentle traction on the umbrella Hakko needle and cystectomy performed. Culdotomy closed	Diameter of ovarian cysts, outcome of culdotomy, operating time for culdotomy, blood loss during culdotomy, complications of culdotomy, CRP on day 3 postoperatively and histology	Mean maximum cyst diameter: 6.9 cm ± 2.1 Operating time for culdotomy: <10 min Blood loss during culdotomy: <10 ml No complications including rectal injury. Mean CRP on day 3 postoperatively: 1.47 ± 1.25 Histology: 18 teratomas, 11 serous cystadenomas, three endometriomas and four mucinous cystadenomas	Culdotomy with the use of umbrella Hakko needle (Culdotomy 2U), provides simple, safe and reliable method. Has the potential to make vaginal ovarian cystectomy more accessible to both patients and gynecologists	[16]
10. Baekelandt (2017) Imelda Hospital, Bonheiden, Belgium	To demonstrate a new approach for performing an ovarian cystectomy via *trans*-vaginal NOTES (vNOTES) as alternative for a laparoscopic ovarian cystectomy	Interventional nonrandomized trial	Dorsal lithotomy position, 2.5 cm posterior colpotomy. The POD was opened and a vNOTES port inserted transvaginally. Pneumoperitoneum created, ovarian cyst identified. Conventional endoscopic instruments and endoscope inserted through vNOTES port. Ovarian cortex incised over the cyst with cold scissors. Cyst dissected from cortex with blunt and sharp dissections. Cyst removed through colpotomy in endobag. vNOTES port removed and colpotomy sutured	Success of the procedure without abdominal scars. Measures: Ovarian cyst diameter, operating time, length of stay and visual analog scale score	Procedure successful in all patients (14/14) 100%. No complications. Fertility spared in all case. All patients were discharged within 30 h – 9/14 discharged within 12 h	Benign ovarian cysts can be treated by vNOTES through posterior colpotomy. It is a new, less invasive approach, which can improve patient comfort and better cosmetic results	[17]

CT: Computed tomography; EBL: Estimated blood loss; LAVOC: Laparoscopically assisted vaginal ovarian cystectomy; LOC: Laparoscopic ovarian cystectomy; NAOC: Natural orifice transluminal endoscopic surgery assisted ovarian cystectomy; NOTES: Natural orifice transluminal endoscopic surgery; POD: Pouch of Douglas; SD: Standard deviation; TVUS: *Trans*-vaginal ultrasound; US: Ultrasound; VOC: Vaginal ovarian cystectomy.

### Outcomes of interest & methodological quality assessment

These included OT, length of stay, intra- or postoperative complications, estimated blood loss (EBL), postoperative pain score and rate of cyst spillage. Emphasis was placed on the surgical technique used in each study, particularly of the entry in the peritoneal cavity via the posterior fornix. As mentioned previously, because of the scarcity of relevant studies, nonrandomized studies were also included for the review. For this reason, formal methodological quality assessment of the selected studies was not performed. The above are all summarized in Tables 2 and 3.

## Results

### Search strategy & study selection

A total of 278 records were screened. The publications screened dated from 1966 to 2017, but the oldest paper included for the final review dated from 2008 [9]. Following an initial screen, 240 studies were excluded due to the title alone and 38 abstracts were retained and examined. From those, 26 abstracts were excluded. They were deemed to be irrelevant to the research question (e.g., ovarian cystectomy was not performed through the vaginal route, not available in English, were animal studies or duplicates). Two papers were included after snowball strategy of other relevant studies [10,11]. Of the 14 full text publications examined, ten met the inclusion criteria [5,9–17]. Of the four studies that were excluded, three were unavailable in English and one was a duplicate study. An overview of the search results and screening process is summarized in the study flow diagram (Figure 1).

#### Patient characteristics

The ten studies involved a total of 525 patients, 226 of which underwent VOC via different techniques, while 299 served as controls and had conventional LOC. All studies excluded cases with a possible histological diagnosis of malignant or borderline ovarian tumors. Such exclusion was achieved radiologically by TVUS and, where appropriate MRI and computed tomography (CT), as well as biochemically by checking CA125 in serum. Participants had either simple or complex (albeit benign) ovarian masses such as endometriomas and dermoids, were premenopausal and wished to retain their fertility. Some authors specifically reported suspected adhesional disease and/or obliteration of POD either radiologically or clinically as exclusion criteria [9,13]. One study only included cases of benign ovarian cysts over 10 cm in diameter [14]. Where direct comparison was made between VOC and LOC, there were no significant differences in age and BMI between the two groups. The above are presented in Table 2.

#### Surgical techniques

Five different surgical approaches have been described in the selected studies. Variations were observed in the technique of entering the POD (blindly, ultrasound [US] or laparoscopic guidance) and the technique of ovarian cystectomy (either by exteriorization of the cyst through the vagina or endoscopic cystectomy). These techniques are summarized below:

- Simple VOC: A posterior culdotomy is performed which is further extended digitally to access the ovarian cyst. The cyst is exteriorized vaginally, or aspirated while in the POD before being exteriorized and subsequently enuclated, preserving the healthy ovarian tissue [5].
- Simple VOC with US guidance: A transvaginal US (TVUS) probe with an introducer is placed in the vagina. A needle passed through the introducer is used to puncture the posterior vaginal fornix and enter the POD [9] or directly enter the cyst [11,16] under US guidance. A balloon is inflated, allowing safe extension of culdotomy, after which the cyst is exteriorized and enuclated as per simple VOC.
- Laparoscopically-assisted VOC: Three-port laparoscopy to allow drainage of cyst contents. Posterior culdotomy is performed laparoscopically, after which the collapsed cyst is exteriorized vaginally and enuclated as above. This technique was used for cysts over 10 cm in diameter [14].
- Transvaginal and laparoscopic VOC: Single umbilical laparoscopy at 10 mmHg of CO_2_ insufflation guides vaginal culdotomy. Two trocars inserted via the posterior fornix and graspers used to perform cystectomy endoscopically. The specimen is retrieved vaginally using an endopouch [10].
- Vaginal NOTES: Posterior culdotomy is performed and three trocars are introduced in the POD. Two graspers and a scope are used through the three trocars and after pneumoperitoneum is created, the cyst is either enuclated and retrieved using an endopouch [15,17] or aspirated endoscopically following which it is exteriorized. Enuclation is performed similar to simple VOC [13].

#### Main findings

Ovarian cystectomy through the vaginal route was associated with reduced postoperative pain and higher satisfaction levels when compared with LOC in two retrospective case–control studies [5,13,15]. Similarly, time to return to work was demonstrated to be significantly reduced in VOC when compared with LOC [5]. Loss of income and productivity due to delayed return to work was calculated to £648 and £1869 for VOC and LOC, respectively [5]. Conversely, when a cost analysis was performed by Wang *et al.*, who compared outcomes of ovarian cystectomy via NOTES versus laparoscopy, no significant difference was observed [13]. Overall, no complications have been reported aside from one case of rectal injury requiring defunctioning colostomy. That was a case of severe endometriosis with obliteration of the POD when culdotomy was performed without US guidance [5].

In all but one case, cystectomies have been completed through the vaginal route, where conversion to laparoscopy was required to achieve hemostasis [11]. Spillage seems to have been avoided or reduced in the selected studies. One case–control study by Yoong *et al.*, involving 49 patients (28 treated vaginally and 21 laparoscopically) reported a significant reduction of cyst spillage (6 vs 35%; p < 0.001) [5]. Furthermore, another case–control study evaluating the feasibility of NOTES, reported draining of the cyst contents under direct vision, before exteriorizing the cyst and dissecting it off the ovary [13].

The mean OT varied between different technique of VOC. Yoong *et al.* reported a mean OT of 91.7 min with simple VOC versus 78 min with LOC (p < 0.001) [5]. On the contrary, Wang *et al.* reported a reduction in OT (38 vs 54 min; p < 0.001) with NOTES versus conventional LOC [13]. Tanaka *et al.* did not report any significant difference in the OT between the two approaches [11]. Finally, Wang *et al.*, who performed laparoscopic-assisted VOC for large ovarian masses (10–27 cm) and Ding *et al.*, who performed NOTES, reported mean OT of 62 and 74 min, respectively [14,15].

Five studies reported their EBL [5,10,13–15]. Yoong *et al.* reported a statistically significant increase in EBL in the simple VOC group compared with LOC (116 vs 95 ml; p < 0.001) [5]. Wang *et al.* also demonstrated a statistically significant increase in the EBL between NOTES and LOC (31 vs 21 ml), p = 0.028 [13]. These differences, however, are of no clinical significance. Moreover, Tanaka *et al.* reported a 5% incidence of dyspareunia and postcoital bleeding a month after VOC, which completely resolved at the 2-month follow-up [12].

Table 3 provides more details on the study characteristics, the different surgical techniques used and results.

## Discussion

This review presents the current evidence on the effectiveness of ovarian cystectomy through the vaginal route. The number of studies is limited, which prevents us from drawing definitive and reliable conclusions. However, these preliminary findings demonstrate that VOC could provide a safe and feasible alternative option to the conventional laparoscopic approach. More, large-scale studies will, however, need to be performed before reliable conclusions can be drawn. Only ten studies have been published on this topic, indicating that this approach has not been widely adopted by gynecologists.

Various techniques have been described for the treatment of benign ovarian cysts through the vaginal route, ranging from a simple VOC to laparoscopic-assisted VOC and transvaginal NOTES. Each technique requires different skill sets as well as novel equipment. For example, Tanaka *et al.* have developed culdotomy techniques using a TVUS probe with an introducer for needles, guide wires and catheters [9,11,12].

Accessing the peritoneal cavity safely through the POD and subsequently locating and exteriorising the ovary through a confined surgical field are the main challenges of ovarian cystectomy through the vagina. Tanaka *et al.* performed TVUS intraoperatively, allowing them to safely enter the POD via a posterior culdotomy and access the affected ovary [9,11,12]. Unguided posterior culdotomy, on the other hand, resulted in a rectal injury requiring major corrective surgery and prolonged hospitalization in a case of severe endometriosis [5]. From the abovementioned, it appears that US guidance improves the safety of VOC by ensuring that there is no bowel adherent to the posterior aspect of the uterus. Furthermore, TVUS has the potential of enhancing visualization and localization of the ovaries during an otherwise blind procedure. The disadvantage of Tanaka *et al.*’s technique is that TVUS guidance requires specialist equipment including a *trans*-vaginal probe that can receive guide wires and catheters, which are expensive and not readily available in most hospital settings [9,11,12].

Other approaches – described above – such as laparoscopic-assisted VOC, transvaginal-laparoscopic VOC and vaginal NOTES, involve pneumoperitoneum and abdominal incisions, which defeat the purpose of managing benign ovarian cysts vaginally to minimize postoperative pain and improve recovery. Furthermore, they involve conventional and/or specialist laparoscopic equipment, which are associated with increased operative costs. The main challenge of vaginal NOTES is associated with the restriction and conflict between the instruments during single-port surgery [7]. Moreover, the need to perform surgery from the opposite end of the patient compared with conventional laparoscopy requires different hand–eye coordination and ergonomic skills [18].

An increased incidence of bladder injury has been described in vaginal adnexal surgery, particularly in cases of previous cesarean section [19]. However, this is a concern in extensive/advanced NOTES such as hysterectomy [19]. No bladder injury was reported in the studies where ovarian cystectomies were performed vaginally as the peritoneal cavity was entered through the POD. The ovaries were then immediately accessed without too much manipulation and operating in the anterior pelvic compartment, namely, the uterovesical fold.

Identifying cases at risk of intrapelvic adhesions and endometriosis can be achieved by thorough clinical history and physical examination assessing the mobility, shape of uterus and the presence of endometriotic nodules in the POD and uterosacral ligaments. Furthermore, the use of the International Ovarian Tumour Analysis rules should guide clinicians distinguish benign from borderline and malignant ovarian tumors during preoperative TVUS [20]. Cases suspicious of borderline or malignant ovarian tumors should not be offered the vaginal approach, as the minimal visibility of the rest of the pelvis and abdomen will prevent adequate surgical staging of the disease.

Moreover, preoperative TVUS is the first-line imaging modality for the diagnosis of pelvic endometriosis with high specificity and sensitivity demonstrated in the case of endometriomas [21–24]. Recent evidence suggests that adhesions can be accurately evaluated by real-time dynamic TVUS, using the sliding sign technique. This can determine whether the uterus and ovaries glide freely over the posterior and anterior organs and tissues [25].

### Proposed clinical application

Transabdominal US (TAUS) guidance has been described in other gynecological procedures such as complex hysteroscopic surgery for uterine fibroids and septoplasty [26–29]. The authors of these papers have found that intraoperative US assisted in safe entry in the endometrial cavity and ensured complete resection of the disease with no complications such as rectal or bladder injury. Ma *et al.* performed TAUS-guided transvaginal hydrolaparoscopy in women with subfertility [30]. TAUS guidance allowed safe entrance in the POD including cases with retroverted uterus. The authors reported no complications with TAUS guidance, while in the comparison group (transvaginal hydrolaparoscopy without TAUS guidance), there were three cases of bowel perforation and one case of uterine injury [30].

We, therefore, advocate the use of TAUS guidance during posterior culdotomy, and access and retrieval of the affected ovary. An US machine with a *trans*-abdominal probe is readily available in a modern hospital setting and does not require further specialized equipment or additional costs. From a practical perspective, it allows the surgeon to operate vaginally without having additional equipment (e.g., a TVUS probe and wires) directly into their operating field. TAUS can be performed by a gynecology trainee with adequate experience and when necessary with guidance from the operating surgeon while the patient is in the lithotomy position.

**Figure 2.**
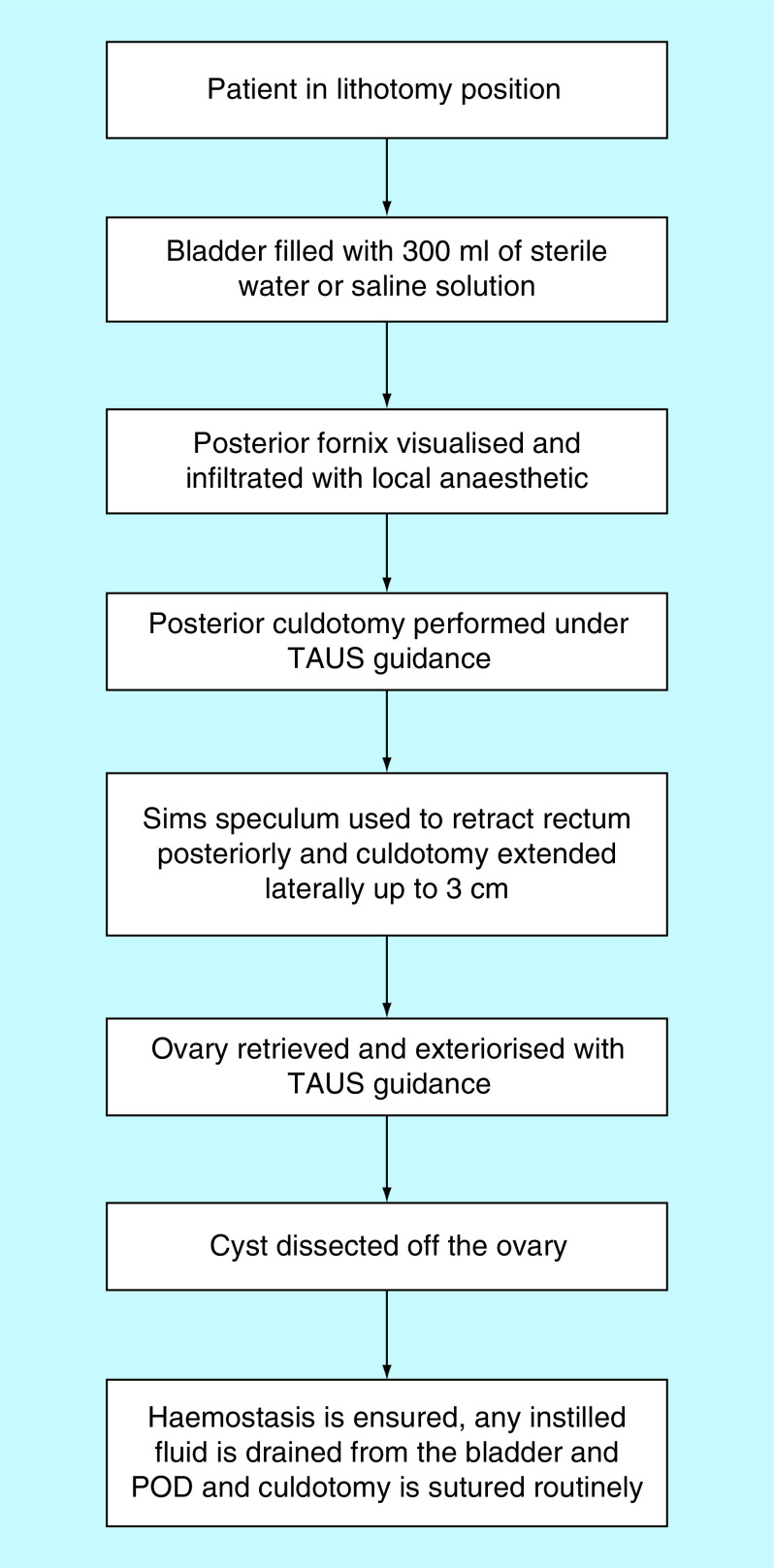
Transabdominal ultrasound during vaginal ovarian cystectomy. POD: Pouch of Douglas; TAUS: Trans-abdominal ultrasound.

For better sonographic evaluation of the uterus, the urinary bladder can be instilled with sterile water or saline solution up to 300 ml and the catheter clamped. In cases of suspected adhesions during preoperative TVUS or from the clinical history, the POD can be instilled with sterile water or saline up to 500 ml via the cervical OS and through the fallopian tubes. Fluid in the POD acts as a contrast medium and optimizes sonographic views by separating the posterior fornix from the rectum, allowing safe posterior culdotomy and minimizing the risk of bowel injury [26–29]. Following infiltration of local anesthetic with adrenaline to the posterior fornix, a 1-cm incision is made. The presence of fluid draining will reassure the surgeon of the correct site of the culdotomy. Alternatively, prior to posterior culdotomy, infiltration of the posterior fornix and aspiration of saline will confirm entry in the POD. Insertion of a Simm’s speculum through the incision can assist in retracting the rectum posteriorly away from the surgical field. The surgeon can extend the incision laterally using their fingers up to 3 cm to allow access and insertion of instruments. The ovary can be retrieved under TAUS guidance and exteriorized before the cyst is dissected off the healthy ovarian tissue. Doppler US can be used to identify pelvic side wall vasculature and minimize vascular injury. At the end of the procedure, any instilled fluid is drained from the bladder and POD. Our proposed clinical application of TAUS-guided VOC is summarized in Figure 2.

### Limitations

This was a thorough review evaluating the safety and feasibility of ovarian cystectomy through the vaginal route. Various techniques and study designs have been described. In addition, a small number of studies and patients have been evaluated in this review which precludes us from drawing reliable conclusions at this stage. However, the findings are promising and provide justification for larger, appropriately powered and standardized studies (ideally randomized-controlled trials) in order to be able to draw definitive conclusions.

Most of the procedures described are relatively novel and therefore, the surgeons performing them may have been at an early phase in their learning curve. In addition, US technology and operators’ scanning skills have significantly evolved over the past decade. These statements suggest that the role of intraoperative US may not be fully appreciated with the current improved technology and skillset. By and large, the studies assessed in this review, described procedures performed by experienced surgeons. It would be interesting to evaluate surgical outcomes of VOC with and without intraoperative US according to surgeons’ experience and capability, to determine which surgeons would benefit the most from it.

Finally, some studies used specialized equipment and US probes not readily available to most hospital settings, including specialized vaginal US probes [10,12,13]. Therefore, the proposed surgical techniques cannot be easily replicated by surgeons around the world. On the other hand, our proposed technique utilizes standard US probes and equipment that are universally accessible in a modern gynecological setting. A proof-of-concept study, followed by large-scale trials will need to be performed to evaluate this proposed technique.

## Conclusion

VOC is an alternative minimally invasive technique for the treatment of benign ovarian cysts in appropriately selected cases based on their risk of intra-pelvic adhesions or endometriosis. Current evidence is promising in terms of patient safety, satisfaction and effectiveness but more studies will need to be conducted before reliable conclusions can be drawn. A variety of techniques have been described in the literature, some requiring specialized equipment. Our proposed modified technique offers an approachable and feasible option that will need to be evaluated in appropriately powered clinical trials.

## Future perspective

As the search for alternative, cost-effective and minimally invasive procedures for the management of benign ovarian cysts ensues, we anticipate more experimentation and publications in this topic. This review should therefore act as a point of reference for future research on the subject. Moreover, the improved skills in US scanning should contribute to better selection of patients preoperatively. In addition, intraoperative US as described in our novel approach could be utilized to potentially reduce the risk of visceral injury when entering the cul-de-sac and thus further promote the vaginal approach.

**Executive summary**

**Background**

- Vaginal ovarian cystectomy (VOC) refers to the management of ovarian cysts via a culdotomy to access the pouch of Douglas.
- Although a well-described procedure, it has never gained wide acceptance among gynecologists.
- We review the current literature to assess the role of VOC as another minimally invasive alternative to laparoscopic ovarian cystectomy.

**Methods**

- Thorough the literature search was conducted through various electronic databases.
- Hand-searching through the references of relevant studies was also conducted.
- All study designs were eligible for this review.
- Outcomes of interest included operating time, length of stay, complications, costs, etc.

**Results**

- Ten studies were included for this review involving a total of 525 patients.
- Various surgical techniques have been used by the different studies, whereby ovarian cysts were removed vaginally.
- Overall, the authors reported favorable outcomes with VOC in regard to: postoperative pain, safety, time to return to work.
- Other outcomes such as operating time and estimated blood loss were inconclusive when VOC was compared with laparoscopic ovarian cystectomy.

**Discussion**

- Overall the findings are promising and provide justification for appropriately powered studies before reliable conclusions can be drawn.
- VOC could be performed in appropriately selected cases.
- Preoperative transvaginal ultrasound plays an important role in case selection.
- Intraoperative transabdominal ultrasound can be a useful adjunct in VOC, particularly in reducing the risk of rectal injury during culdotomy.
- A proof-of-concept study should be carried out in the near future.

**Conclusion & future perspective**

- VOC as an alternative minimally invasive option should be explored further to assess its feasibility, safety and cost–effectiveness.
